# P-1295. Use of Large Language Models Does Not Improve Model Performance for Antimicrobial Resistance Prediction in Community- and Hospital-Onset Gram Negative Sepsis

**DOI:** 10.1093/ofid/ofaf695.1483

**Published:** 2026-01-11

**Authors:** Alison M Hixon, Hanyang Liu, Michael J Durkin, Jennie H Kwon, Andrew Atkinson, Chenyang Lu, Maria Cristina Vazquez Guillamet

**Affiliations:** Washington University, St. Louis, MO; Washington University, St. Louis, MO; Washington University School of Medicine, St. Louis, MO; Northwestern University , Chicago , IL; Washington University School of Medicine, St. Louis, MO; Department of Computer Science and Engineering, Washington University in St. Louis, St. Louis, Missouri; Washington University in St. Louis, St.Louis, Missouri

## Abstract

**Background:**

Treatment of sepsis requires prompt initiation of appropriate empiric antibiotics. Coverage for Gram-negative bacilli (GNB) is decided based on risk factors for resistant organisms, as documented in both structured and unstructured data in the electronic health record. We hypothesized that adding large language models (LLMs) that can decode clinical narratives would improve performance of deep learning models in predicting antimicrobial resistance in GNB sepsis.

**Figure:**
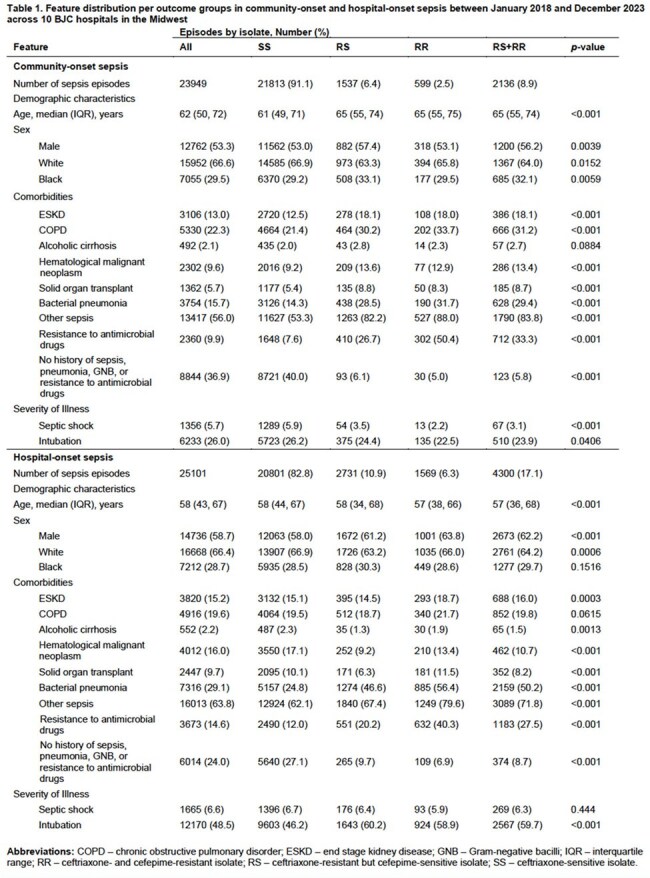

**Figure:**
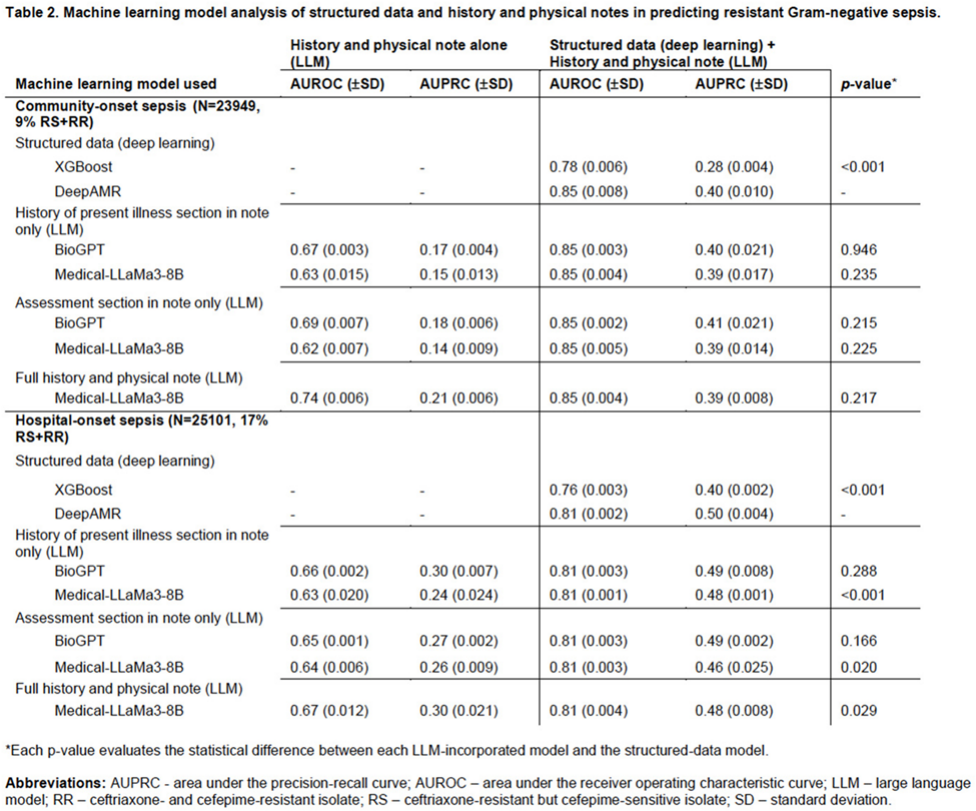

**Methods:**

This retrospective cohort analysis included all adult patients who met CDC sepsis criteria admitted to 10 BJC hospitals from 1/2018 to 12/2023. Sepsis episodes were classified as community-onset (< 48hrs from hospitalization) or hospital-onset. GNB were stratified as ceftriaxone-susceptible (SS), ceftriaxone-resistant but cefepime-susceptible (RS), and ceftriaxone- and cefepime-resistant (RR). Culture negative or non-GNB sepsis were labeled SS. Deep learning models (XGBoost, DeepAMR) were developed using all available structured data (e.g., demographics, vitals, labs). LLMs (BioGPT, Medical-LLaMa3-8B) were developed on full or partial history and physical exam notes. Models were assessed using area under the receiver operating characteristic curve (AUROC) and area under precision-recall curve (AUPRC).

**Figure:**
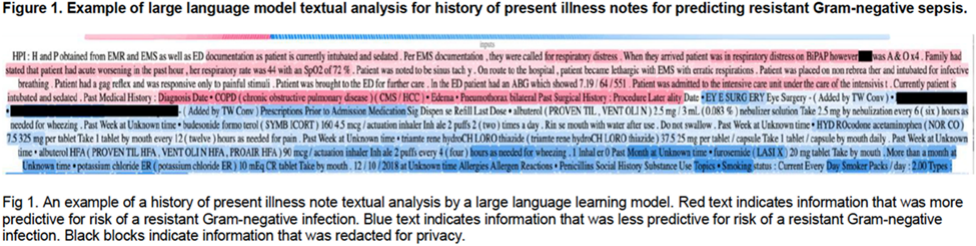

**Results:**

A total of 29,517 patients were included, for 23,949 community-onset and 25,101 hospital-onset sepsis episodes with RS+RR GNB in 9% and 17%, respectively (Table 1). Relevant features of structured data models included history of prior infections and resistant organisms. DeepAMR was the best performing deep learning model with AUROC 0.85/AUPRC 0.40 and AUROC 0.81/AUPRC 0.50 for community- and hospital-onset sepsis, respectively (Table 2). The best performing LLM was Medical-LLaMa3-8B on full notes (Figure 1), with AUROC 0.74/AUPRC 0.21 and AUROC 0.67/AUPRC 0.30 for community- and hospital-onset sepsis, respectively. Combining deep learning and LLMs did not significantly improve the prediction of resistant infections compared to deep learning alone.

**Conclusion:**

Structured medical data provided the highest prediction of resistant GNB infections in sepsis. More analysis is needed to understand why the unstructured data did not improve model predictive performance.

**Disclosures:**

Maria Cristina Vazquez Guillamet, MD, Bionano: Stocks/Bonds (Public Company)|Charisma: Stocks/Bonds (Public Company)|Ocugen: Stocks/Bonds (Public Company)

